# Perceptions of breast cancer risk after breast density notification in a population-based screening program

**DOI:** 10.1007/s10549-025-07662-1

**Published:** 2025-03-06

**Authors:** Dorinda ‘t Hart, Ross Marriott, Jennifer Stone

**Affiliations:** School of Population and Global Health, University of Western Australia, 35 Stirling Highway, Crawley, Perth, WA 6009 Australia

**Keywords:** Breast cancer, Personal risk, Perceived risk, Risk factors, Breast density, Latent variable analysis

## Abstract

**Background:**

Despite increasing evidence to support risk-based breast cancer screening, individuals’ understanding of personal risk is not well understood. This study compares women’s perceptions of risk to their estimated risk, and examines factors associated with perceived risk, including breast density notification, within a population-based screening program.

**Methods:**

A survey of 5784 women measured their perceived risk via three questions: a number from 0 to 100 (*numeric)*, a category from very low to very high (*verbal)*, a comparative category relative to an average woman (*comparative)*. Descriptive analyses assessed correlations between perceived risk variables and estimated risk (using the Gail Model), and modelled relationships using K-fold cross-validation. A Graded Response Model was used to obtain an index of unobserved (latent) overall perceived risk from the three questions. Multivariable modelling was used to investigate factors associated with overall perceived risk.

**Results:**

Most participants perceived themselves as being at neither high nor low risk, although perceived risk was higher than estimated risk, on average. All three perceived risk measures were positively correlated with each other and with estimated risk. Overall perceived risk was weakly associated with estimated risk (adjusted *R*^2^ = 0.12). Women who received multiple breast density notifications, were younger, or had a family history, perceived their risk as higher relative to respective reference groups. Those who identified as Asian perceived their risk as lower than those who identified as European/Caucasian.

**Conclusion:**

Individuals’ understanding of breast cancer risk is poor. New strategies are needed to improve education and awareness of personal risk.

**Supplementary Information:**

The online version contains supplementary material available at 10.1007/s10549-025-07662-1.

## Introduction

Breast cancer is the leading cause of cancer-related deaths internationally [1]. Population-based breast cancer screening increases early detection which improves treatment options and saves lives [2, 3]. There is increasing evidence to suggest that more personalised risk-based screening could improve the effectiveness and efficiency of mammographic screening by identifying and targeting those who could benefit most from risk-reducing strategies, increased screening interval and/or supplemental modalities [4–6]. However, very little is known about what individuals understand about their personal breast cancer risk and what factors inform their perceived risk.

Risk perception refers to individuals’ subjective judgments about the likelihood of negative outcomes such as injury, illness, disease, and death [7]. Perceived risk is an established motivator of cancer screening [8–10] and, therefore, increased understanding of individuals’ perceptions of breast cancer risk is integral to developing effective risk communication messages regarding risk-based screening and the importance of routine screening participation. In general, factors that influence risk perception in women include age, sociodemographic variables, and culture [11, 12]. With relation to breast cancer, this list extends to include personal and family history of the disease and potentially, breast density notification [13].

How much individuals know and understand about risk is a key component of risk perception. For example, public knowledge regarding breast density, the radiographic appearance of epithelial and stromal tissue on a mammogram, and its association with reduced sensitivity of mammography appears to be understood [8–10]. However, knowledge and awareness of breast density as a risk factor are generally poor [13–15]. Disparities in knowledge and awareness of breast density as a risk factor are also often intensified across ethnicities and more socioeconomically disadvantaged groups [13, 16].

This study uses three questions from the literature to assess perceived risk [17] within the population-based BreastScreen Western Australia (WA) program. We estimate an overall measure of perceived risk, derived using latent variable modelling, and examine its correlation with estimated risk (using the Gail model) and its association with other factors.

## Methods

### Source of participants

Ethics and governance approval was obtained from King Edward Memorial Hospital’s Human Research Ethics Committee (#2017046EW RGS000474) and The University of Western Australia (RA/4/20/4178).

The BreastScreen WA program screens approximately 130,000 women per year and is free for all women over the age of 40 years. Breast density notification is binary, where women only receive notification if a radiologist flags that they have dense breasts.

### Survey data

As previously reported, the survey was developed from validated questions from the literature and significant consumer engagement [18, 19]. Demographic information included year of birth and postcode (from which an education and occupation index were derived using data from the Australian Bureau of Statistics). Breast density notification status was self-reported and validated by the type of survey link provided to participants [18]. Respondents were categorized into three groups: controls, those notified for the first time, and those notified multiple times.

Information to inform the Gail model Breast Cancer Risk Assessment Tool [20] included ethnicity, age, age at menarche, age at first live birth, number of first-degree relatives with breast cancer, number of previous breast biopsies, and atypical hyperplasia status [21]. The absolute risk of developing breast cancer was calculated using the “absolute.risk” function of the BCRA package in R [22] with 75 as the projection age.

Participants were asked to rate their lifetime risk of developing breast cancer on a scale of 0–100 (henceforth, numeric), to rate their chance of developing breast cancer on a 5-point Likert scale from very low to very high (henceforth, verbal), and to rate their chance of developing breast cancer compared to the average women their age on a 3-point Likert scale of less likely, about as likely, or more likely (henceforth, comparative).

### Recruitment

As previously reported, the survey was administered via Qualtrics to all women who attended BreastScreen WA between 21 November 2017 and 19 April 2018 who received a routine results letter and had an email address on record. Emails were sent to 30,566 eligible women and the response rate was 23.5% [18]. Breast density notification status was known for each participant. Otherwise, responses were anonymous unless women opted to provide their contact details for future contact [19].

### Descriptive analysis

Descriptive statistics were estimated by breast density notification status. Plots were constructed to inspect pairwise relationships between each of the perceived risk variables (numeric, verbal, comparative) and with estimated Gail risk score. Spearman’s rank correlation (ρ) was used to quantify correlations for ordinal variables.

K-fold cross-validation was used to evaluate how well each of the perceived risk variables predict the estimated Gail risk score, on their own or in combination. A dummy-coded variable identifying if the numeric score was exactly 50% or not was included as an additional model predictor to determine if this common response was important for explaining estimated risk, additive to numeric. The full set of additive combinations of the survey variable predictors comprised 11 candidate models, which were each fitted to 9 randomly allocated folds of the dataset (training data). Estimated risk scores were cube root and log transformed, respectively, to correct for positive skewness. The predictive ability of models was ranked using the mean-squared error (MSE) of model predictions within the held-out fold (test data). The coefficient of determination, *R*^2^, and the adjusted *R*^2^, ${R}_{\text{adj}}^{2}$, were estimated for the model fits to the training data. The MSE and out-of-sample *R*^2^, ${R}_{\text{oos}}^{2}$ [23] were estimated for model predictions to test data. Cross-validations were repeated, each time using a different fold as test data, which resulted in 10 sets of the *R*^2^, ${R}_{\text{adj}}^{2}$, MSE, and ${R}_{\text{oos}}^{2}$ statistics, and the means of these were presented for each model. The equation for the best predicting model is provided in Appendix 1 in Supplemental Material.

### Latent variable modelling

Latent variable modelling was used to estimate an unobserved overall perceived risk index from the observed perceived risk variables or “manifest variables.” Graded response models (GRMs) were used to model ordinal manifest variables [24]. Although the numeric variable is continuous, 31.9% of survey participants responded exactly 50%, a common phenomenon that may reflect uncertainty or dichotomy of a breast cancer diagnosis rather than a specific value [17]. Accordingly, the numeric variable was transformed into an ordinal variable with quintile cut points (ignoring values of exactly 50%).

Two versions of the analysis were completed. In GRM analysis 1, the GRM was fitted to all manifest variables at once (GRM 1). In GRM analysis 2, separate models were fitted to manifest variable data for (i) respondents providing a numeric score of exactly 50% (GRM 2a) and (ii) respondents who provided a numeric score that was not exactly 50% (GRM 2b). Model fit diagnostics (residual plots, marginal χ^2^ contingency table tests of model fit, item response curves, coefficient estimates) were evaluated for each of these GRMs. The normally distributed factor scores for each response pattern were rescaled to overall perceived risk scores by: (i) multiplying by the standard deviation and adding the mean of the numeric variable (Scaling 1); and (ii) using the same method as (i) but excluding exact 50% responses from the mean and standard deviation calculations (Scaling 2). These rescaled values were then mapped back to the respondent data to obtain an index of overall perceived risk.

Each index of overall perceived risk was plotted against the estimated Gail risk for each respondent to visually assess agreement and departures from unity. A linear regression was fitted to model the association of cube-root-transformed estimated risk with overall perceived risk. The Box–Cox method selected the cube-root transformation as being suitable to obtain normally distributed residuals.

### Determinants of perceived risk

Univariable and multivariable regression was used to investigate the variation in the index of overall perceived risk explained by breast density notification, age, ethnicity, regionality, education and occupation index, and family history of breast cancer. All factors were modelled as categorical terms except for age, which was modelled as a continuous predictor using a restricted cubic spline, with internal knots pre-specified at the 27.5th, 50th, and 72.5th percentiles and boundary knots at the 5th and 95th percentiles, following the recommendations of Harrell (2015) [25].

Marginal effect plots with 95% confidence intervals were constructed to graphically depict significant effects. All analyses were done in R version 4.4.1 [26].

## Results

### Descriptive analysis

Table 1 presents a summary of demographic characteristics of all survey respondents, showing controls, respondents notified for the first time and respondents notified multiple times. Those notified for the first time (henceforth, first timers) were younger than controls and women who had been notified multiple times. In all three groups, the majority of the women were Caucasian/European (86.7–88.7%), lived in a major city (80.4–83.3%) and were generally in the higher quintiles on the education and occupation index. More than two thirds of respondents did not have a relative with breast cancer.

**Table 1:** Characteristics of respondents by breast density notification status

Characteristics	Controls (*n* = 3274)	Notified for first time (*n* = 1313)	Notified multiple times (*n* = 1194)
Mean age at survey (SD)	60.9 (8.2)	57.6 (9.1)	59.8 (8.0)
*Ethnicity*			
Caucasian/European	2841 (86.8)	1138 (86.7)	1059 (88.7)
Asian	103 (3.2)	62 (4.7)	40 (3.4)
Aboriginal or Torres Strait Islander	13 (0.4)	2 (0.2)	4 (0.3)
Other	100 (3.1)	48 (3.7)	35 (2.9)
Missing	217 (6.6)	63 (4.8)	56 (4.7)
*ARIA*^*a*^* (%)*			
Major city	2632 (80.4)	1094 (83.3)	967 (81.0)
Inner regional	259 (7.9)	83 (6.3)	88 (7.4)
Outer regional	149 (4.6)	46 (3.5)	53 (4.4)
Remote	164 (5.0)	63 (4.8)	68 (5.7)
Very remote	33 (1.0)	14 (1.1)	11 (0.9)
Missing	37 (1.1)	13 (1.0)	7 (0.6)
*Education and occupation index*^*b,c*^* (%)*			
1 (lowest)	368 (11.2)	130 (9.9)	128 (10.7)
2	599 (18.3)	209 (15.9)	220 (18.4)
3	517 (15.8)	184 (14.0)	159 (13.3)
4	658 (20.1)	271 (20.6)	233 (19.5)
5 (highest)	1095 (33.5)	506 (38.5)	447 (37.4)
Missing	37 (1.1)	13 (1.0)	7 (0.6)
*Number of relatives with breast cancer (%)*			
0	2187 (66.8)	987 (75.2)	800 (67.0)
1	574 (17.5)	186 (14.2)	223 (18.7)
> 1	145 (4.4)	40 (3.0)	66 (5.5)
Unknown	147 (4.5)	36 (2.7)	49 (4.1)
Missing	221 (6.8)	64 (4.9)	56 4.7)

*SD* standard deviation

^a^Accessibility/remoteness index of Australia (ARIA) scores

^b^Scores are on a scale from 1 to 5, where 1 indicates the lowest 20% of the population in the state (least education and occupation opportunities) and 5 indicates the highest 20% of the population in the state (most economic resources and most education/occupation opportunities)

^c^Index of education and occupation based on Western Australian state rankings

Table 2 summarizes responses from the three perceived risk questions. On the numeric scale, respondents rated their lifetime risk of developing breast cancer between 33.5% and 35.9% which is well above the national statistic 1 in 8 (12%) and the estimated risks from 5.8 to 6.9%. The mode (most common) response was 50%. Removing the respondents who reported exactly 50% reduced the mean lifetime risk to 25.5–28.3%, lowest in first timers. On the verbal scale, a higher percentage of first timers perceived themselves at being at very low to neither high nor low risk compared to controls (83.1% vs. 80.2%, respectively) and compared to those notified multiple times (78.4%). On the comparative scale, the percentage of respondents who reported their breast cancer risk as being about the same or much lower/less likely than other women their age was similar between first-timers and controls (84.9% and 85.4%, respectively), but lower in those who had received multiple notifications (81.9%).

**Table 2:** Responses to survey questions regarding perceived risk of breast cancer and the estimated Gail risk score, by breast density notification status

	Controls (*n* = 3274)	Notified first time (*n* = 1313)	Notified multiple times (*n* = 1194)
*What do you think your chance is of developing breast cancer in your lifetime? (Numeric)*			
Mean numerical (0–100%) (SD)	35.3 (22.1)	33.5 (21.7)	35.9 (21.1)
Median numerical	40	30	40
Mean numerical (0–100%) with 50% removed (SD)	27.6 (24.3)	25.5 (22.5)	28.3 (22.8)
Median numerical with 50% removed	20	20	20
Missing	230	84	67
*How would you rate your chance of developing breast cancer? (Verbal)*			
Very low	586 (17.9)	206 (15.7)	153 (12.8)
Moderately low	829 (25.3)	376 (28.6)	291 (24.4)
Neither high nor low	1211 (37.0)	510 (38.8)	492 (41.2)
Moderately high	407 (12.4)	142 (10.8)	188 (15.8)
Very high	34 (1.0)	18 (1.4)	16 (1.3)
Missing	207 (6.3)	61 (4.7)	54 (4.5)
*Overall, how do you think your chance of developing breast cancer compares to the average woman your age? (Comparative)*			
Much lower/less likely	434 (13.3)	280 (21.3)	199 (16.7)
About the same	2345 (71.6)	841 (64.1)	778 (65.2)
Much higher/more likely	282 (8.6)	130 (9.9)	164 (13.7)
Missing	213 (6.5)	62 (4.7)	53 (4.4)
Calculated risk using Gail model	2941	1210	1111
Mean estimated to 75 years	5.8 (3.9)	6.6 (3.8)	6.9 (4.8)
Median estimated	5.1	6.2	5.9

**Fig. 1:**
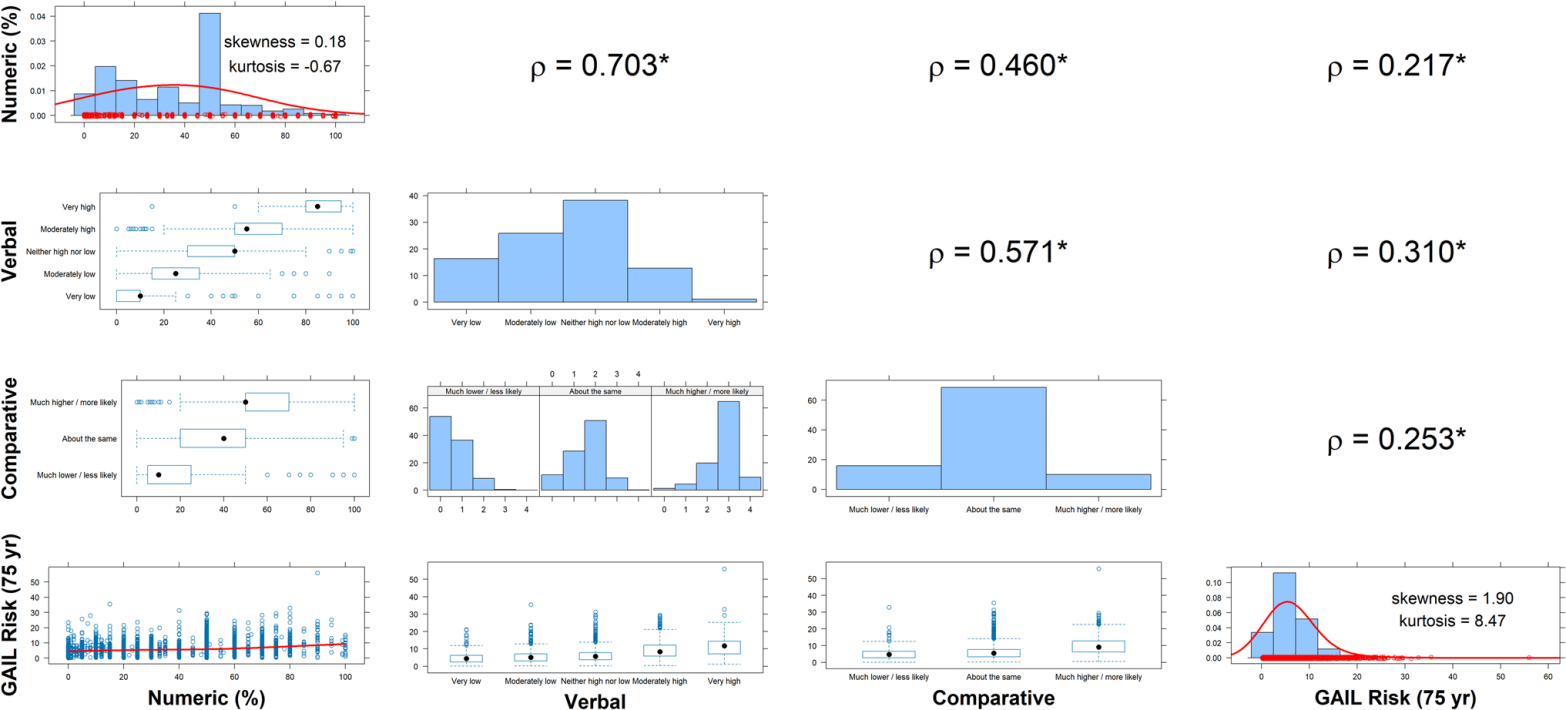
Pairwise plots of associations between observed variables of perceived and calculated breast cancer risk. * denotes significant departure of Spearman’s rank correlation from zero (*p* < 0.05). GAIL risk score is calculated up to 75 years (yr) of age

Spearman’s rank correlations demonstrated significant positive pairwise associations between all 3 perceived risk variables, and between each of these and the Gail risk score (Fig. 1). The estimated Gail risk score was positively skewed (Skewness = 1.90), so a cube-root transformation was applied.

Ten-fold cross-validations were used to rank 11 candidate models comprised of terms for each perceived risk variable, on its own or in combination with others, for predicting the Gail risk score (Table 3). The model including all three perceived risk variables was identified as the best for predicting estimated risk, although predictions were relatively weak (out-of-sample *R*^2^ = 0.13).

**Table 3:** Results from tenfold cross-validation of candidate models for predicting Gail risk score from observed perceived risk variables

Model	Fit to training data		Predict to test data		Overall ranking
Dependent variable: cube root (Gail risk score to 75 years)	*R*^2^	${R}_{\text{adj}}^{2}$	Mean squared error	${R}_{\text{oos}}^{2}$	
N + N50 + V + C	0.137	0.135	0.140	0.131	1 (Best)
N + V + C	0.136	0.135	0.140	0.131	2
V + C	0.133	0.132	0.140	0.129	3
N + N50 + V	0.121	0.119	0.142	0.116	4
N + V	0.12	0.119	0.142	0.116	5
V	0.118	0.117	0.142	0.115	6
N + N50 + C	0.109	0.108	0.144	0.105	7
N + C	0.108	0.107	0.144	0.104	8
C	0.091	0.09	0.147	0.087	9
N + N50	0.059	0.059	0.152	0.057	10
N	0.055	0.055	0.153	0.053	11 (Worst)

*N* Numeric; ‘N50’ = {Numeric = 50%, Numeric is not 50%}; *V* Verbal; *C* Comparative; ‘${R}_{\text{adj}}^{2}$’ adjusted *R*^2^; ‘${R}_{\text{oos}}^{2}$’ out-of-sample *R*^2^

### Latent variable modelling

In GRM analysis 1, the fitted model demonstrated lack of fit (GRM 1; See Appendix 2 in Supplemental Material). In GRM analysis 2, fitting separate GRMs to data for respondents providing a numeric score of exactly 50% (GRM 2a), versus all other respondents (GRM 2b), resulted in improved fit (based on the distributions of residuals with factor scores and item information curves (Supplementary Figure S1). Therefore, results are presented for indices derived from GRM analysis 2 (GRM 2a and GRM 2b).

**Fig. 2:**
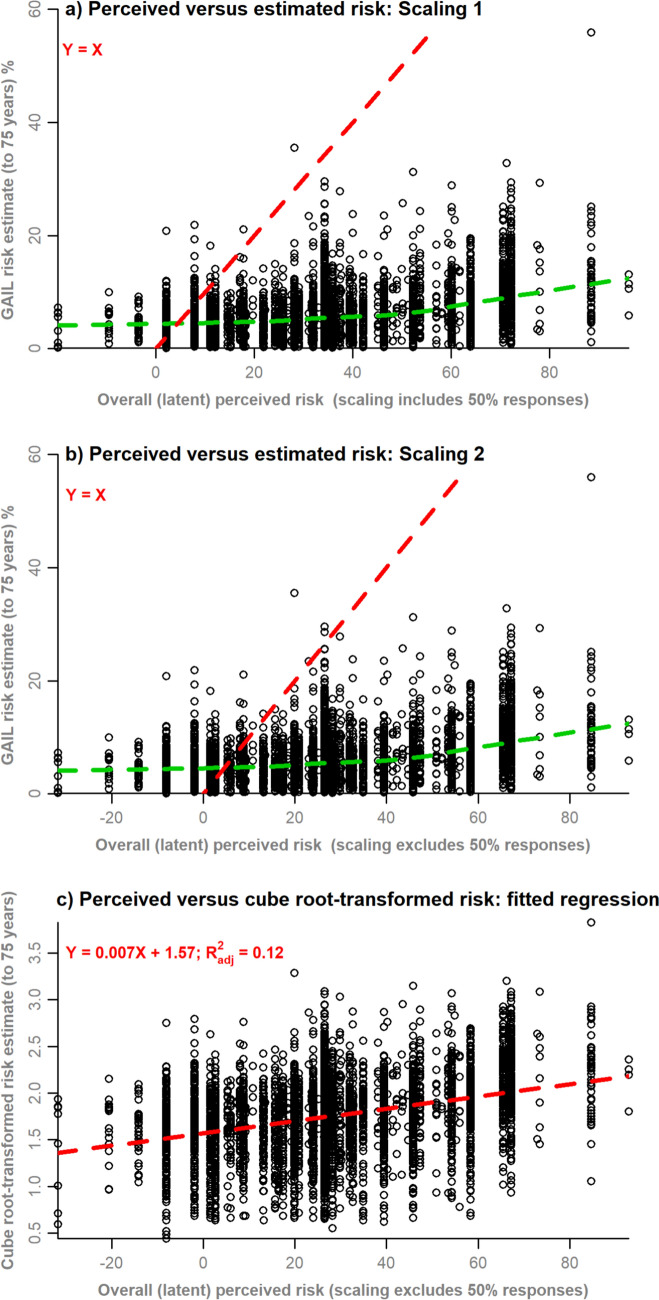
Overall perceived risk calculated from GRM analysis 2 plotted against estimated risk **a** using Scaling 1; **b** using Scaling 2; **c** regressed against cube-root-transformed risk

Scaling method 2 resulted in closer approximations, on average, to the Gail-estimated risk scores (Fig. 2a, b), although both scaling methods resulted in some unrealistic negative values. A non-linear local regression through the points (green dashed lines) demonstrates marked departure from the line of agreement (red dashed lines) and shows that Gail-estimated risk is generally lower than the majority (84.8%) of overall perceived risk scores, but on average higher for the lowest (i.e. < 4%) values of overall perceived risk (Fig. 2b). However, there is much variability in the Gail-estimated risk score with overall perceived risk and still weakly associated after transformation (adjusted *R*^2^ = 0.12) (Fig. 2c).

### Factors associated with perceived risk

**Fig. 3:**
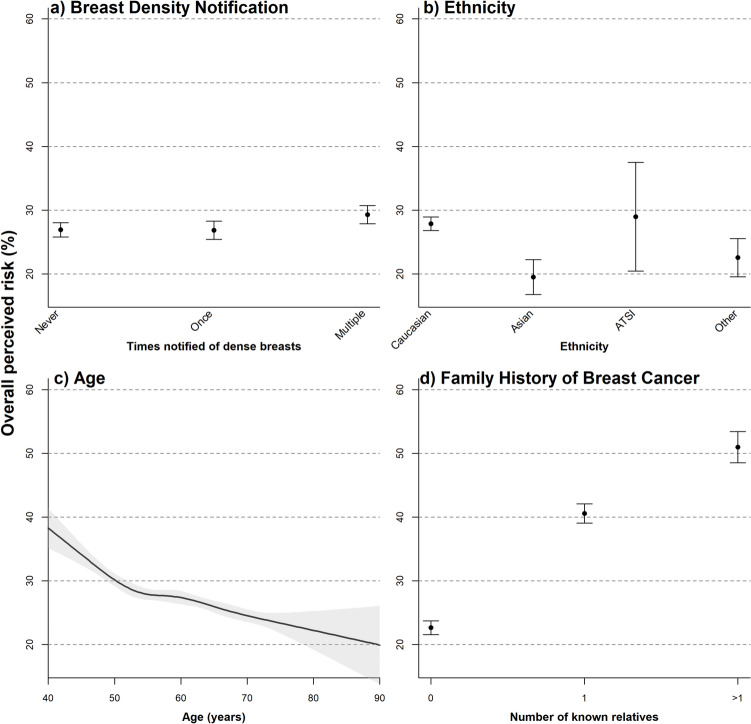
Marginal effect plots of significant predictors from the multivariable analysis. Error bars show 95% confidence intervals of estimated marginal means from the multivariable model, after controlling for all other terms in the model

Univariable regression analysis demonstrated a significant effect of dense breast notification on overall perceived risk, as determined using estimates from GRM analysis 2 (GRM 2a, 2b) with Scaling method 2 (Table 4). In multivariable modelling, the effects of dense breast notification, age, ethnicity, and family history of breast cancer on overall perceived risk were significant (Table 4). The estimated effect of dense breast notification was only slightly attenuated, after controlling for all other predictors. Overall perceived risk was estimated to be 2.38% higher for women who had received multiple notifications (EMM = 29.3%, CI = 27.9%, 30.7%) as compared with controls (EMM = 26.9%, CI = 25.8%, 28.0%; Fig. 3a). Although modelled using a non-linear spline, overall perceived risk demonstrated a monotonic decline with age, after controlling for all other factors (Table 4; Fig. 3c). There was a pronounced effect of family history on overall perceived risk, being 17.9% higher for respondents with 1 known relative, and 28.3% higher for respondents with > 1 known relative, as compared to those with no known relatives with history of breast cancer (Table 4; Fig. 3d).

**Table 4:** Univariable and multivariable analyses of overall perceived risk

Model	Predictor	Effect^a^	Standard error	*P*-value (term)
Univariable	Dense breast notification			
	Never (ref)	0		< 0.001
	Once	−0.29	0.71	
	Multiple times	+ 3.18	0.73	
Multivariable	Dense breast notification			
	Never (ref)	0		< 0.001
	Once	−0.06	0.65	
	Multiple times	+ 2.38	0.66	
	Age^b^	−1.53	0.59	< 0.001
	Ethnicity			
	Caucasian (ref)	0		< 0.001
	Asian	−8.38	1.35	
	Aboriginal and Torres Strait Islander	+ 1.10	4.33	
	Other	−5.32	1.48	
	ARIA			
	Major city (ref)	0		0.93
	Inner Regional	+ 0.48	1.02	
	Outer Regional	+ 1.04	1.33	
	Remote	+ 0.11	1.29	
	Very remote	+ 0.79	2.6	
	Advantage and disadvantage index			
	Quintile 1 (ref)	0		0.48
	Quintile 2	−0.78	1.2	
	Quintile 3	+ 0.63	1.22	
	Quintile 4	+ 0.86	1.22	
	Quintile 5	+ 0.31	1.15	
	Family history of breast cancer			
	0 relatives (ref)	0		< 0.001
	1 relative	+ 17.92	0.66	
	> 1 relative	+ 28.33	1.20	

^a^Effect size is the mean difference from the reference level (change in overall perceived risk (%), calculated using estimates from GRM analysis 2 with Scaling method 2)

^b^Effect size is the change in overall perceived risk arising from an increase in 1 standard deviation about the mean age (from 55.6 to 64.1 years)

## Discussion

This study shows that most individuals do not fully understand their personal breast cancer risk, particularly regarding breast density. Most participants perceived themselves as being at neither high nor low risk or at moderately low risk. Overall, women who had increasing number of relatives with breast cancer correctly perceived that their risk was higher, and Asian women perceived their risk as lower (compared to Caucasian/European). However, younger women perceived their risk to be higher than older women, contrary to the established association between increasing age and breast cancer risk. Women who had been notified they had dense breasts for the first time did not perceive themselves at higher risk compared to controls, but those who had been notified multiple times did perceive their risk as being higher. This suggests that knowledge of breast density as a risk factor for breast cancer may increase over time. Combined, these findings suggest that improved education about individual risk is needed, not just breast density-associated risks.

All three perceived risk measures were positively correlated with each other and with estimated risk, using the Gail model. However, the index of overall perceived risk was weakly associated with estimated risk (adjusted *R*^2^ = 0.12). Although overall perceived risk was higher than Gail-estimated risk for most respondents, there is much unexplained variation about the fit of the model. The high unexplained residual variation prevents us from making any strong conclusion concerning the nature of how perceived risk under- or over-estimates Gail-estimated risk, except that estimates of perceived risk do not make very good predictions of Gail-estimated risk.

From the literature, most women do not have accurate perceptions of their personal breast cancer risk, often optimistically biased but influenced by family history, age, education, and race/culture [12, 27–30]. Consistent with this study, an Australian study found that women tended to overestimate the incidence of breast cancer in the population, whilst perceiving themselves to be at low to average risk [10]. They also found that most women are cognizant of the impact of family history on their personal breast cancer risk [10]. Our result that younger women perceived their risk to be higher compared to older women, is not uncommon, with many studies reporting that younger women report higher levels of perceived risk [10, 12, 28–30]. Finally, we found that women who identified as Asian perceived themselves at lower risk compared to European/Caucasian women. This is consistent with a study that found that Asian Pacific women were less likely to overestimate their risk compared to white women [31]. The same study found no association between perceived risk and education, consistent with the lack of association with the education and occupation index used in this study.

In terms of breast density, this study showed that women notified they had dense breasts for the first time did not perceive themselves at a higher risk of breast cancer, compared to controls. This is consistent with the literature that suggests that women lack knowledge of breast density as a breast cancer risk factor, even after notification [13, 14, 32, 33]. Tran et al. showed that women who were aware of their breast density and/or had a good level of knowledge on breast density, were more likely to perceive their risk as high [8]. In this study women who were notified multiple times did perceive their risk as higher, suggesting that multiple notifications may improve breast density awareness and knowledge. A randomized controlled trial of a video intervention to improve women’s knowledge of their breast cancer risk and breast density (compared to the standard breast density notification letter) demonstrated that knowledge of Gail-estimated breast cancer risk and measured breast density can be improved [12]. Women appear to be keen to learn more about their personal breast density and what that means as a breast cancer risk factor [13, 33]. Further evidence of the feasibility and acceptability of personalized interventions to improve knowledge and awareness of personal risk are warranted.

Strengths of this study include its large size and generalisability within a population-based screening program. It also provides a control group of women who have not been notified of their breast density to make direct comparisons. The latent variable modelling of three commonly asked questions to assess perceived risk is, as far as we can tell, quite novel, and enabled direct comparison of two continuous measures of risk, perceived and estimated. A potential limitation is that whilst the Gail model has been shown to be a validated risk assessment tool in Australian screening populations [34], the Gail model used in this analysis did not include breast density to calculate estimated risk [20], potentially reducing the accuracy of the estimated risks.

In summary, this study adds to the body of evidence that most women do not have accurate perceptions of their personal breast cancer risk and suggests that repeated messaging may improve perceptions of breast density-associated risks. Understanding risk perception is important in health and communication as it informs which risks individuals care about and how they deal with them [7]. This is particularly important as population-based screening programs consider moving towards more personalised breast cancer screening, instead of the current “one-size-fits-all” approach. Future research should consider extending beyond increasing knowledge of breast density and its associated risks, particularly as AI-derived mammographic measures are expected to surpass breast density’s discriminatory power as a predictor of breast cancer risk [35]. Improved understanding of mammogram-associated risk scores will pave the way for more personalized risk-based screening and more effective screening outcomes. Co-designed strategies/interventions that personalize information to educate individuals about their risk [32] could significantly improve early detection of breast cancer through increased screening participation.

## Supplementary Information

Below is the link to the electronic supplementary material.

Supplementary file1 (DOCX 61 KB)

## Data Availability

No datasets were generated or analysed during the current study.
