# Supplementary material for: Perceptions of breast cancer risk after breast density notification in a population-based screening program
Source: Breast Cancer Res Treat. 2025 Mar 6;211(2):455–65. doi: 10.1007/s10549-025-07662-1 (PMC12006211; doi:10.1007/s10549-025-07662-1)
Supplement: Supplementary file 1 — Supplementary file1 (DOCX 61 KB) [file 10549_2025_7662_MOESM1_ESM.docx]

**Appendix 1. Best predictive model for cube-root transformed estimated risk**

The best predictive model for cube-root transformed estimated risk (Gail model lifetime risk to 75 years) was estimated as:

$$\sqrt[3]{\text{risk}}=1.57+8.6\times{10}^{-6}N-0.015N50+0.098V_{\text{mod\_low}}+0.184V_{\text{not\_high\_or\_low}}+0.338V_{\text{mod\_high}}+0.487V_{\text{very\_high}}+0.098C_{\text{same}}+0.098C_{\text{higher}}$$

Where: *N* is Numeric score (0-100%); *N*50 is a dummy variable coded 1 if *N* was exactly 50%, 0 otherwise; *V* variables are dummy-coded for Verbal responses of moderately low, not high or low, moderately high, or very high; *C* variables are dummy-coded for Comparative responses of about the same or much higher/more likely.

**Appendix 2: LVM Analysis**

*LVM Analysis 1.*

A significant lack of model fit was demonstrated from Chi-squared tests of the two-way marginal distributions for frequencies of the 3 ordinal perceived risk variables. This lack of fit is reflected in the apparent non-linear trend of residuals with fitted values (Supplementary Figure S1a).

*LVM Analysis 2.*

GRMs were fitted separately to perceived risk data: (i) for individuals providing a Numeric score of exactly 50% (*GRM 2a*); and (ii) for those who provided a Numeric score that was not 50% (*GRM 2b*). GRM 2a was fitted to only the Verbal and Comparative variables (since all values of Percent Categories were identical), whereas GRM 2b was fitted to all 3 perceived risk variables (including Percent Categories). No lack of model fit was demonstrated from Chi-squared tests of the two-way marginal distributions for GRM 2a but a significant lack of fit was demonstrated for GRM 2b. Lack of fit for GRM 2b appears to be reflected in more positive residuals at the tail of the distribution (i.e., factor scores > 1.5; Supplementary Figure S5). Nevertheless, model diagnostics demonstrate a general improvement in fit from the stratified approach, as compared with that of GRM 1.


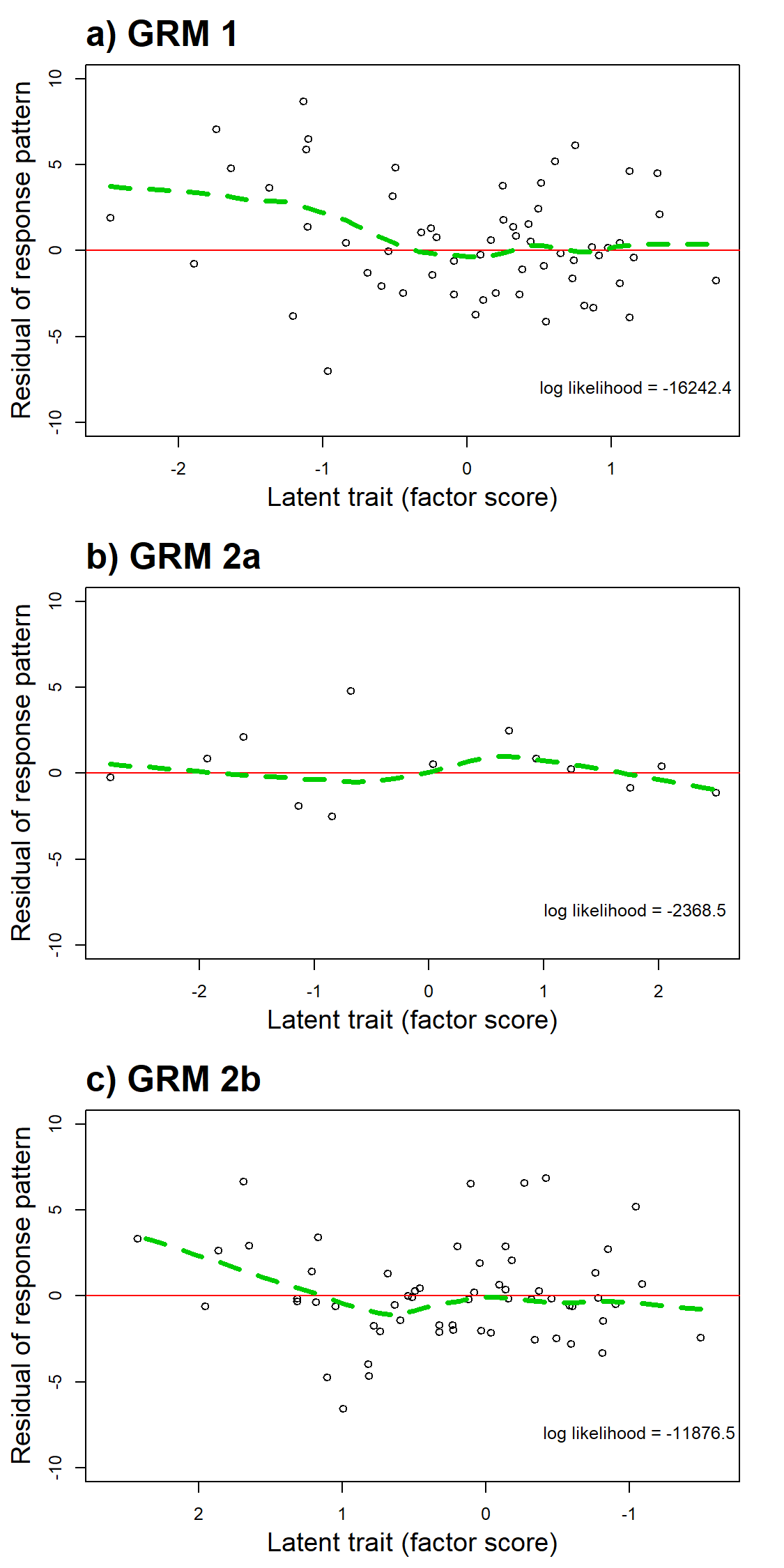


**Supplementary Figure S1.** Chi-square residuals with factor scores for modelled response patterns by Graded Response Models (GRMs). Plots represent GRM fits to a) all 3 ordinal perceived risk variables (manifest variables) for all individuals (GRM 1) and separately to data for individuals: b) providing a Numeric score of exactly 50%, with Verbal and Comparative as the manifest variables (GRM 2a); and c) providing a Numeric score other than exactly 50%, with all 3 perceived risk variables as the manifest variables (GRM 2b). Green dashed lines show fitted local regressions demonstrating the trend in residuals, as distributed with model-estimated values of the latent trait. For GRM 2b, the x-axis scale has been reversed to ensure that factor scores are comparable with those estimated for GRM 1 and GRM 2a, in terms of the unobserved overall perceived risk.
